# Efficacy of telbivudine in Taiwanese chronic hepatitis B patients compared with GLOBE extension study and predicting treatment outcome by HBV DNA kinetics at Week 24

**DOI:** 10.1186/1471-230X-12-178

**Published:** 2012-12-13

**Authors:** Chao Wei Hsu, You Chen Chao, Chuan Mo Lee, Ting Tsung Chang, Yi Cheng Chen

**Affiliations:** 1 Liver Research Unit, Chang Gung Memorial Hospital, Chang Gung University College of Medicine, 199 Tung Hwa North Road, Taipei, 105, Taiwan; 2 Buddhist Tzu Chi General Hospital, Taipei Branch, Taiwan; 3 Liver Research Unit, Chang Gung Memorial Hospital, Chang Gung University College of Medicine, Kaohsiung, Taiwan; 4 National Cheng Kung University Hospital, Tainan, Taiwan

**Keywords:** Chronic hepatitis B, HBV DNA kinetics, Roadmap concept

## Abstract

**Background:**

The aims of this study were to compare results from a Taiwanese sub-study of the GLOBE 2303 telbivudine study and evaluate the HBV DNA kinetics.

**Methods:**

Forty-one Taiwanese patients were treated for an additional 2 years with telbivudine. Efficacy endpoints were the same as the GLOBE study. The correlations of reductions in HBV DNA levels at Week 24 were evaluated.

**Results:**

All 7 HBeAg-positive patients with undetectable HBV DNA levels at Week 24 sustained this response at Year 4 with rates of ALT normalization 71%, HBeAg seroconversion 57%, and cumulative resistance 0%. Out of 16 HBeAg-negative patients with undetectable HBV DNA levels at Week 24, 11 (78%) sustained this response at Year 4 with rates of ALT normalization 83% and cumulative resistance 8.7%. There were significant correlations between reductions of DNA of ≥5 log_10_ copies/mL at Week 24 with maintained PCR negativity at Years 2–4 and a lack of resistance at Year 2.

**Conclusions:**

Long-term telbivudine efficacy in Taiwanese patients was comparable to the GLOBE 2303 study. A reduction in HBV DNA levels by ≥5 log_10_ copies/mL at Week 24 represented the optimal cut-off point, which may predict favourable outcomes in patients with high baseline HBV DNA levels.

**Trial registration:**

ClinicalTrials.gov Identifier: NCT00142298 (http://clinicaltrials.gov/).

## Background

Chronic hepatitis B (CHB) infection is an important risk factor for the development of liver failure, liver cirrhosis, and hepatocellular carcinoma. The prevalence of hepatitis B virus (HBV) in Taiwan (>10%) is the highest in Asia, with 76% of chronic hepatitis patients being infected with HBV [1]. Although the implementation of a mass immunization program against HBV has brought a dramatic reduction in the prevalence of the carrier state, there were approximately 2.4 million HBV carriers in Taiwan in 2003 [2]. Genotypes B and C are the predominant HBV strains in Taiwan, whereas genotypes A, D, E, and F are the predominant genotypes in Caucasian patients [3]. A more frequent relapse after stopping lamivudine therapy has been observed, especially if consolidation therapy after HBeAg seroconversion is too short [4].

The Phase III GLOBE trial demonstrated the superior efficacy of telbivudine over lamivudine over a period of two years in a population of 1370 patients who had both HBeAg-positive and HBeAg-negative CHB [5,6]. The Phase IIIb extension study involved an additional two years of telbivudine therapy with continued monitoring of therapeutic responses, and further demonstrated a higher rate of viral suppression along with better durability and favourable safety profile of telbivudine compared to lamivudine [7,8]. The roadmap concept uses a treatment algorithm based on patient outcomes at Week 24 to maximize benefits from long-term therapy against HBV and minimize long-term drug resistance [9,10]. In the current management regimen for CHB, the residual HBV DNA level is considered to be a better marker of therapeutic outcome and the emergence of resistance than baseline HBV DNA levels. However, in patients with high HBV DNA baseline levels (≥9 log_10_ copies/mL), it is difficult to achieve absolute HBV DNA non-detectability within 24 weeks of treatment given that the average HBV DNA reduction of even the most potent NAs is only 6–7 log_10_ copies/mL per year [11]. The importance of evaluating pre-treatment characteristics such as baseline HBV DNA levels versus early on-treatment responses in predicting long-term treatment outcomes in CHB has not been definitively established.

In the present study, we analyzed the efficacy of telbivudine in a homogeneous population of Taiwanese patients subanalysis to the GLOBE 2303 study. In addition, we evaluated the correlation of viral load kinetics (exact decrement in HBV DNA levels) at Week 24 with the efficacy in treatment Years 1–4 in telbivudine-treated Taiwanese CHB patients.

## Methods

### Study design and patients

A total of 59 Taiwanese patients in the telbivudine arm and 56 Taiwanese patients in the lamivudine arm from the intent to treat (ITT) population of the GLOBE study were included in this study. Signed informed consent was obtained from the patients and the treatment was approved by the joint institutional review board (NV-02B-022) and the department of health (0940305529) in Taiwan. Four patients from the telbivudine arm discontinued treatment before the end of the 2-year study due to efficacy (achieved HBeAg seroconversion), and 10 patients from the lamivudine arm withdrew before the end of the 2-year study due to efficacy (n = 1) and protocol deviations or adverse events (n = 9). In total, 55 patients in the telbivudine arm and 46 patients in the lamivudine arm completed treatment in the GLOBE study. Of these, 48 patients in the telbivudine arm and 36 patients in the lamivudine arm who had polymerase chain reaction (PCR)-undetectable or PCR-detectable serum HBV DNA levels and did not show genotypic resistance to the two drugs entered the Phase IIIB extension 2303 study. Considering the significant superiority of telbivudine over lamivudine demonstrated by the international GLOBE trial, all patients who entered the 2303 study were treated with telbivudine for an additional two years due to ethical concerns. A total of nine patients withdrew from the study (7 in the telbivudine arm and 2 in the lamivudine-switch arm) due to protocol deviations and/or adverse events. In total, 34 (18 HBeAg-positive and 16 HBeAg-negative) of the 36 patients from the lamivudine-switch arm and 41 (16 HBeAg-positive and 25 HBeAg-negative) of the 48 patients from the telbivudine arm completed the 2303 study. In the present study, the 4-year efficacy and on-treatment durability of the 41 Taiwanese patients from the telbivudine arm of the GLOBE trial who completed the 2303 study were analyzed.

To study the HBV DNA kinetics, the data obtained from all of the 34 HBeAg-positive telbivudine-treated patients completing the 2303 study were analyzed. The baseline characteristics of the Taiwanese patients in the GLOBE trial, 2303 study, and HBV kinetics study are shown in Table 1.

**Table 1:** Baseline characteristics of the Taiwanese population in the GLOBE trial, 2303 study, and HBV DNA kinetics analysis

**Characteristic**	**GLOBE trial**		**Study 2303**		**HBV DNA reduction kinetics study HBeAg-positive patients from GLOBE**		
	**HBeAg- positive (n = 16)**	**HBeAg- negative (n = 25)**	**HBeAg- positive (n = 16)**	**HBeAg- negative (n = 25)**	**≤5 log_10_ copies/mL at Week 24 (n = 8)**	**>5 log_10_ copies/mL at Week 24 (n = 26)**	**P value**
**Age (years)**							
Median (min–max)	27.5 (19–53)	45.0 (20–63)	29.5 (21–55)	47.0 (22–65)	38.0 (20–45)	27.5 (19–56)	
Age range, n (%)							
<30	9 (56.3)	1 (4.0)	8 (50.0)	1 (4.0)	3 (37.5)	15 (57.7)	
30–50	6 (37.5)	19 (76.0)	7 (43.8)	17 (68.0)	5 (62.5)	8 (30.8)	0.223
>50	1 (6.3)	5 (20.0)	1 (6.3)	7 (28.0)	0 (0)	3 (11.5)	
**Gender**							
Male, n(%)	11 (68.8)	21 (84.0)	11 (68.8)	21 (84.0)	5 (62.5)	19 (73.1)	0.566
Female, n(%)	5 (31.3)	4 (16.0)	5 (31.3)	4 (16.0)	3 (37.5)	7 (26.9)	
**Weight**							
Median (min–max)	67.5 (48–95)	68.0 (42–95)	69.5 (51–92)	65.5 (43–91)	61.5 (49–73)	67.5 (38–95)	
**HBV genotype, n(%)**							
B	13 (81.3)	17 (68.0)	13 (81.3)	17 (68.0)	5 (62.5)	16 (61.5)	
C	3 (18.8)	8 (32.0)	3 (18.8)	8 (32.0)	3 (37.5)	10 (38.5)	0.961
**HBV DNA (log**_**10**_**copies/mL)**							
Median (min–max)	10.2 (7–13)	7.2 (4–13)	2.5 (2–10)	2.2 (2–5)	8.9 (6–10)	10.2 (8–15)	
**ALT (U/L)**							
Median (min–max)	134.5 (51–288)	110.0 (40–499)	27.5 (10–272)	29.0 (15–63)	92.0 (53–231)	149.0 (43–767)	

### Efficacy assessments

Efficacy measures for the analyses were as described in a previous publication [6]. Standardized tests were performed centrally by Quintiles Transnational (Research Triangle Park, NC). Serum HBV DNA levels were quantified using a COBAS® Amplicor HBV Monitor PCR assay (COBAS-AM assay; Roche Molecular Systems, Pleasanton, CA, USA; detection limit, 300 copies/mL). Informed consent was obtained from each patient enrolled in the study. The study was conducted in compliance with the Declaration of Helsinki and in accordance with Good Clinical Practice guidelines and local regulations.

### Statistical analysis

Because only patients treated with telbivudine were included in this analysis, no statistical comparisons between treatment groups were performed. Analyses of the 4-year efficacy results (HBV DNA levels of <300 log_10_ copies/mL, alanine aminotransferase (ALT) normalization (the normal range of ALT was below 48 IU/mL in male patients and 37 IU/mL in female patients), HBeAg loss and seroconversion, and resistance) were based on observed data, and missing data were not imputed. The cumulative HBeAg seroconversion rate was defined as the percentage of HBeAg-positive patients (n = 16) with documented seroconversion at any point during the 4-year treatment period, including patients who subsequently seroconverted. Genotypic resistance was defined as the emergence of treatment-associated resistant mutations identified by direct sequencing of the amplified HBV DNA at baseline and from sera of all patients with serum HBV DNA levels of >3 log_10_ copies/mL at Year 4 (Week 208) [6]. Viral breakthrough was defined as a persistent (two consecutive determinations) on-treatment increase in serum HBV DNA level of >1 log_10_ copies/mL above the nadir level [12].

The correlations of viral load kinetics (exact reduction in HBV DNA) at Week 24 with PCR negativity, ALT normalization, HBeAg seroconversion, and treatment-emergent resistance at Years 1, 2, 3, and 4 of telbivudine treatment were also evaluated. Patients were categorised into three groups according to serum HBV DNA reduction at treatment Week 24: Group 1, ≤5 log_10_ vs. >5 log_10_ copies/mL; Group 2, ≤6 log_10_ vs. >6 log_10_ copies/mL; and Group 3, ≤7 log_10_ vs. >7 log_10_ copies/mL. Stepwise logistic regression analyses were performed to identify variables associated with treatment outcomes. An odds ratio of >1 indicated a direct relationship, whereas an odds ratio of <1 indicated an inverse relationship [8]. A *P* value of less than 0.05 was considered statistically significant.

## Results

### Virological and biochemical responses at Year 4

The efficacy results of continuous telbivudine treatment in the 41 compensated CHB Taiwanese patients, the ITT population of the telbivudine arm of the GLOBE trial analysed in this study, were consistent with those of the worldwide GLOBE trial. In the 4-year worldwide GLOBE study (GLOBE study and extension 2303 study) of telbivudine [13], 79% of the HBeAg-positive patients achieved undetectable HBV DNA levels (<300 log_10_ copies/mL) with 51% HBeAg seroconversion, and 84% of the HBeAg-negative patients achieved undetectable serum HBV DNA levels and 91% achieved ALT normalization at Year 4. In the present subanalysis of the Taiwanese patients, 50% of the HBeAg-positive patients had undetectable serum HBV DNA levels, 56.3% had normalized ALT, and 37.5% and 25% achieved HBeAg loss and seroconversion, respectively, at Year 4. The HBeAg-negative patients achieved better efficacy endpoints: 76% had undetectable serum HBV DNA levels and 83% had normalized ALT at Year 4. In the Taiwanese patients who had undetectable or detectable HBV DNA levels at study entry, genotypic resistance to telbivudine at Year 4 was observed in 43.8% of the HBeAg-positive patients, and 12.5% of the HBeAg-negative patients (Table 2). In Taiwanese treated patients had lower HBeAg seroconversion and higher genotypic resistance rate compare to GLOBE study might be prolonged HBV infection and genotype B and C.

**Table 2:** Efficacy results at year 4 in the telbivudine-treated Taiwanese population of the GLOBE trial

	**All ITT patients**		**Patients with HBV DNA level <300 copies/mL at Week 24**	
**Characteristic**	**HBeAg-positive (n = 16)**	**HBeAg-negative (n = 25)**	**HBeAg-positive (n = 7)**	**HBeAg-negative (n = 16)**
HBV DNA level				
<300 copies/mL, n (%)	8/16 (50.0)	19/25 (76.0)	7/7 (100.0)	11/16 (78.3)
ALT normalization, n (%)	9/16 (56.3)	20/24 (83.0)	5/7 (71.4)	11/15 (86.4)
HBeAg loss, n (%)	6/16 (37.5)	NA	6/7 (85.7)	NA
HBeAg seroconversion, n (%)	4/16 (25)	NA	4/7 (57.1)	NA
Resistance, n (%)	7/16 (43.8)	3/24 (12.5)	0/7 (0.0)	2/23 (8.7)

### Maintained virological responses to telbivudine at 4 years

Seven out of the 16 HBeAg-positive patients who achieved undetectable levels of serum HBV DNA at Week 24 sustained this response (100% response rate) at the end of Year 4. Among the HBeAg-negative patients, 16 patients achieved PCR negativity at Week 24, of whom 11 (78.3%) sustained the response at the end of Year 4. Overall, the proportion of patients achieving ALT normalization and HBeAg loss and seroconversion was higher in the subgroup of patients with undetectable serum HBV DNA levels at Week 24 (Table 2). Undetectable HBV DNA levels at treatment Week 24 were also associated with lower rates of genotypic resistance to telbivudine at Year 4 in the HBeAg-positive (0%) and HBeAg-negative patients (8.7%) (Table 2).

**Figure 1:**
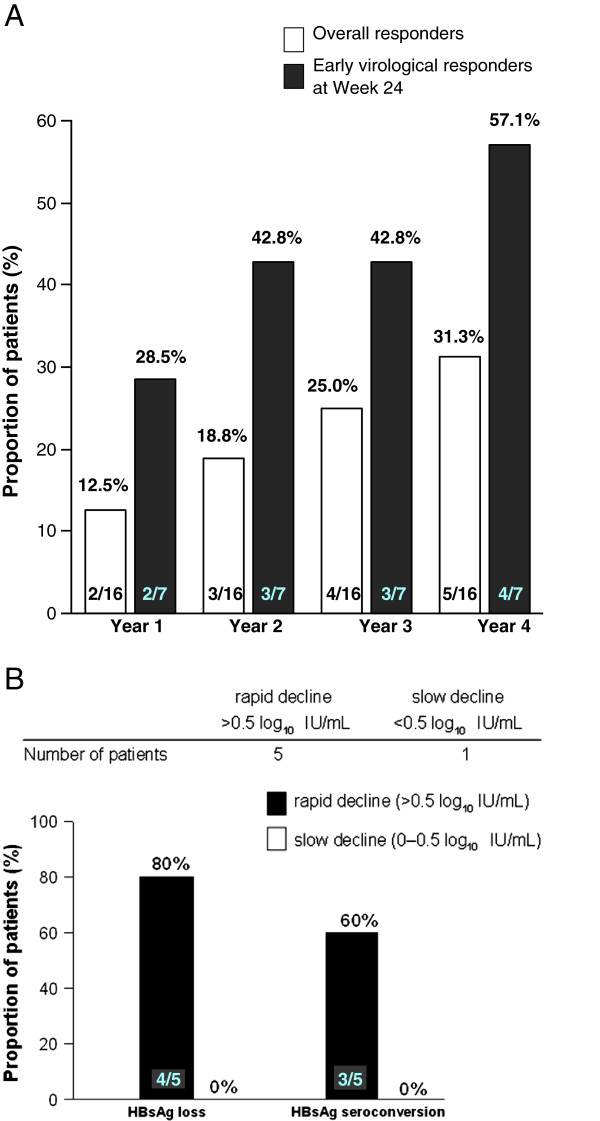
A. Cumulative HBeAg seroconversion rates with continuous telbivudine treatment for 4 years in the ITT population of HBeAg-positive Taiwanese patients. B. HBsAg kinetics during telbivudine treatment.

The rate of cumulative HBeAg seroconversion increased with continued telbivudine treatment from 12.5% at the end of Year 1 to 31.3% at the end of Year 4. In line with other efficacy results, the rate of seroconversion was higher (28.5% at Year 1 and 57.1% at Year 4) in the subgroup of patients with undetectable serum HBV DNA levels at Week 24 (Figure 1A).

### HBsAg kinetics during treatment

In patients with HBeAg-positive CHB, telbivudine treatment significantly and progressively reduced serum HBsAg levels over a 3-year period in the subanalysis of the worldwide GLOBE study [14]. In the present study, six HBeAg-positive CHB Taiwanese patients were enrolled in an HBsAg sub-study of the 4-year GLOBE study. Of these, five patients showed a rapid decline (>0.5 log_10_ IU/mL) and one patient showed a slow decline (<0.5 log_10_ IU/mL) in HBsAg levels at Week 24. Patients who had a rapid decline in HBsAg levels had higher HBsAg loss and seroconversion rates (80% and 60%, respectively) compared to patients with a slow decline (0%), at that time point, their HBV DNA less than 300 copies/mL (Figure 1B). Interestingly, the proportion of patients with a rapid decline in HBsAg levels from baseline to Week 24 was higher in the Taiwanese subanalysis (83.3%) than in the worldwide GLOBE study (33%) [14]. However, HBsAg loss or seroclearance with S-gene mutations has recently been reported [15,16], and an immune escape-like mutant has been proposed. Further studies are necessary for the simultaneous evaluation of HBsAg-negative and HBV DNA levels.

### Correlation between the reduction of HBV DNA levels at Week 24 and treatment outcomes at Year 4

We investigated the viral load kinetics (exact decrement in HBV DNA levels) at Week 24 and the correlations with long-term efficacy endpoints, namely maintained PCR negativity, ALT normalization, HBeAg seroconversion, and treatment-emergent resistance at Years 1, 2, 3 and 4 of continuous telbivudine treatment. The average baseline HBV DNA level of the 34 HBeAg-positive patients in the correlation studies was ~9.6 log_10_ copies/mL. Reductions of 5, 6, and 7 log_10_ copies/mL in HBV DNA levels at Week 24 were compared against post-treatment outcomes at Years 1, 2, 3 and 4. Statistically significant correlations were observed only between HBV DNA reductions of 5 log_10_ copies/mL and maintained PCR negativity (with the exception of treatment Year 1), and between HBV DNA reductions of 5 log_10_ copies/mL and lack of resistance. At two years post-treatment, the odds ratios of an HBV DNA reduction of <5 log_10_ copies/mL at Week 24 for maintained PCR negativity and treatment-emergent resistance were 0.075 and 9.953, with *P* values of 0.0288 and 0.0219, respectively (*P* < 0.05) (Table 3). At Years 3 and 4, the odds ratios for maintained PCR negativity were 0.056 and 0.053, with *P* values of 0.019 and 0.0164, respectively. The odds ratios for resistance at Years 3 and 4 could not be calculated given that the patients who had an HBV DNA reduction of <5 log_10_ copies/mL at Week 24 developed resistance at Years 3 and 4 post-treatment. The higher cumulative telbivudine resistance rates at Years 1, 2, 3, and 4 in patients with an HBV DNA reduction <5 log_10_ copies/mL compared to patients with an HBV DNA reduction >5 log_10_ copies/mL (Table 4) were significantly correlated with HBV DNA reduction levels at Week 24, and further identified an HBV DNA reduction of >5 log_10_ copies/mL at Week 24 as a good predictor of treatment outcomes at four years of continuous telbivudine treatment.

**Table 3:** Predictability of efficacy endpoints for an HBV DNA reduction threshold of ≤5 log_10_ copies/mL vs. >5 log_10_ copies/mL at Week 24

**Endpoints**	**Odds ratio**	**95**% **CI**	***P *value**
**Treatment Year 1**			
Maintained PCR negativity	0.650	(0.119, 3.541)	0.6183
Maintained ALT normalization	0.283	(0.032, 2.530)	0.2586
Maintained HBeAg loss	0.000	(0.000, >999.9)	0.9619
Maintained HBeAg seroconversion	0.000	(0.000, >999.9)	0.9517
**Treatment Year 2**			
Maintained PCR negativity	0.075	(0.007, 0.765)	0.0288
Maintained ALT normalization	0.349	(0.056, 2.174)	0.2592
Maintained HBeAg loss	0.339	(0.032, 3.616)	0.3707
Maintained HBeAg seroconversion	0.368	(0.033, 4.085)	0.4156
Cumulative treatment-emergent resistance	9.593	(1.387, 66.347)	0.0219
**Treatment Year 3**			
Maintained PCR negativity	0.056	(0.005, 0.623)	0.0190
Maintained ALT normalization	0.313	(0.053, 1.855)	0.2010
Maintained HBeAg loss	0.124	(0.012, 1.300)	0.0816
Maintained HBeAg seroconversion	0.313	(0.030, 3.231)	0.3295
Cumulative treatment-emergent resistance	>999.9	(0.000, >999.9)	0.9681
**Treatment Year 4**			
Maintained PCR negativity	0.053	(0.005, 0.584)	0.0164
Maintained ALT normalization	0.195	(0.032, 1.1980	0.0776
Maintained HBeAg loss	0.147	(0.015, 1.4530	0.1009
Maintained HBeAg seroconversion	0.219	(0.022, 2.209)	0.1976
Cumulative treatment-emergent resistance	>999.9	(0.000, >999.9)	0.9681

**Table 4:** Cumulative resistance rates with continuous 4-year telbivudine treatment in HBeAg-positive and HBeAg-negative Taiwanese patients showing an HBV DNA decline of >5 log_10_ copies/mL and ≤5 log_10_ copies/mL

		**HBeAg-positive patients (%)**	**HBeAg-negative patients (%)**
Genotypic resistance to telbivudine	Year 4	43.8%	12.5%
**Predictive factors at Week 24**			
Undetectable/Detectable levels of HBV DNA	Year 4	0%/77.8%	8.7%/100%
Achieved/Non-achieved reduction of 5 log_10_ copies/mL	Year 1	0%/0%	NA
	Year 2	7.6%/75%	NA
	Year 3	15%/100%	NA
	Year 4	15%/100%	NA

## Discussion

In the GLOBE trial and the extended Phase IIIb study (2303) confirmed a good safety and tolerability profile of telbivudine, along with prolonged viral suppression and biochemical responses in HBeAg-positive and HBeAg-negative patients. The present study was a subanalysis of 41 Taiwanese patients (~75% with genotype B and ~25% with genotype C) from the telbivudine arm of the worldwide GLOBE study who successfully completed the 2303 study. The results of the Taiwanese subanalysis were in agreement with the worldwide GLOBE 2303 study, despite differences in genotypes, disease progression, and transmission patterns between the Taiwanese population and other races from the worldwide GLOBE trial. Both studies prove the long-term (4-year) efficacy of telbivudine with maintained virological responses in early responders (PCR-negative at Week 24), favourable ALT normalization in both HBeAg-positive and HBeAg-negative patients, and a high HBeAg seroconversion rate in HBeAg-positive patients. Previous telbivudine studies focussing on Asian patients with CHB have reported efficacy results up to 1, 2 and 3 years in Korea, China and Hong Kong [3,17,18]. In our study presents a more comprehensive and long-term follow-up of telbivudine-treated Taiwanese patients.

The roadmap concept assesses antiviral efficacy at Week 24 to make treatment adjustments based on the decline in HBV DNA levels. At Week 24, the decline in serum HBV DNA levels is further categorised as complete (< 300 copies/mL), partial (300 to 10000 copies/mL), or inadequate (≥ 10000 copies/mL) [19]. However, these thresholds are not applicable to patients with a high viral load at baseline given that the average HBV DNA reduction with even the most potent NAs is only 6–7 log copies/mL in one year of treatment. The present study is the first to characterize HBV DNA reduction kinetics during long-term oral treatment with a direct and highly specific antiviral agent such as telbivudine in order to predict efficacy endpoints, namely maintained PCR negativity, ALT normalization, HBeAg seroconversion, and treatment-emergent resistance. We evaluated a number of HBV DNA reduction cut-off values (5, 6, and 7 log_10_ copies/mL) and identified an HBV DNA reduction of >5 log_10_ copies/mL as a good predictor of effective treatment outcomes at treatment Year 4. HBV DNA reductions of 6 and 7 log_10_ copies/mL did not show a significant correlation with any of the efficacy endpoints. We believe that this approach may be complementary to the widely used roadmap concept in the long-term management of CHB patients, particularly for patients with high baseline levels of HBV DNA (≥9 log_10_ copies/mL). Establishing a definite threshold of HBV DNA reduction in CHB patients with high baseline values may help to identify a subset of patients with effective on-treatment suppression of HBV replication. When complemented with the current roadmap concept, monitoring of on-treatment HBV DNA kinetics may be able to provide better treatment adjustments in patients who are likely to achieve HBeAg clearance and seroconversion during long-term telbivudine therapy.

The European Association for the Study of the Liver guidelines [20], and the roadmap concept proposed by Keeffe et al. [9] suggest the use of serum HBV DNA levels at Week 12 as the first early predictor of treatment efficacy. Week 24 has been proposed as the next earliest predictor of outcomes [21]. In the present study, the reduction in HBV DNA level at Week 24 was significantly correlated with maintained PCR negativity and treatment-emergent resistance. Analysis of the baseline characteristics of the CHB patients from the GLOBE trial identified Week 24, but not Week 12, as the optimal time point to predict treatment outcomes at the end of treatment Year 2 based on the serum HBV DNA levels in both HBeAg-positive and HBeAg-negative telbivudine-treated patients [8]. Serum HBV DNA levels at Week 24 have also been shown to influence long-term outcomes in lamivudine recipients [22] and in lamivudine-resistant adefovir recipients [23], demonstrating its usefulness in the management of CHB patients. However, the predictive value of HBV DNA levels at Week 12 in predicting long-term outcomes has been demonstrated in efficacy studies of adefovir [24]. Additionally, in a 3-year telbivudine efficacy trial conducted in Hong Kong, which included 36 patients from the GLOBE trial, HBV DNA levels of <200 IU/mL at Week 12 were predictive of a higher chance of HBV DNA undetectability and a lower chance of resistance by Year 3. Undetectable levels of HBV DNA at Week 24 was predictive of viral suppression at Year 2 but not at Year 3 [18].

It is worth noting that a higher proportion of patients in the Taiwanese sub-study (83.3%) had a rapid decline in HBsAg levels (83.3%) than in the worldwide GLOBE study (78%). Interestingly, these Taiwanese patients who had a rapid decline in HBsAg had higher HBsAg loss and seroconversion rates (80% and 60%, respectively) than patients with a slow decline (0%). Although these results need to be verified in a larger population of Taiwanese patients, our data show telbivudine to be an effective antiviral agent in the long-term treatment of Taiwanese patients with CHB.

The current guidelines and the US treatment algorithm [19] recommend entecavir and tenofovir as first-line monotherapies for CHB due to their relative potency and high genetic barrier to resistance [25]. However, the Asian-Pacific consensus statement on the management of CHB in 2008 suggests the use of telbivudine in addition to entecavir and tenofovir [26]. Superior efficacy and lower resistance rates of telbivudine over lamivudine have been demonstrated by the worldwide GLOBE and accompanying trials.

## Conclusion

The long-term efficacy and durability of continuous telbivudine treatment demonstrated in our study of Taiwanese patients substantiates the use of telbivudine in the treatment of CHB from an Asian-Pacific perspective and is comparable to the GLOBE 2303 study. Furthermore, the importance of monitoring changes in HBV DNA reduction kinetics (the optimal cut-off level of HBV DNA reduction is ≥ 5 log_10_ copies/mL) to predict the 4-year outcomes of telbivudine treatment may be a novel idea, and can be effectively incorporated in the roadmap algorithm for monitoring patients with high baseline HBV DNA levels of ≥9 log_10_ copies/mL.

## Abbreviations

CHB: Chronic hepatitis B; HBV: Hepatitis B virus; ITT: Intent to treat population; PCR: Polymerase chain reaction.

## Competing interest

The authors have no financial or personal relationships with other people or organizations that could inappropriately influence (bias) their work. Dr. You Chen Chao served at the Tri-Service Hospital, Taipei, but moved to the Buddhist Tzu Chi General Hospital, Taipei, after completion of the study.

## Authors’ contribution

Dr. Chao Wei Hsu contributed to acquisition of the data, analysis and interpretation of the data, statistical analysis and drafting the manuscript. The other authors contributed to establishing the study concept and revision of the manuscript. All authors read and approved the final manuscript.

## Grant support

This study is a subanalysis of the GLOBE 2303 study [6] sponsored by Novartis. There is no additional grant support.

## Pre-publication history

The pre-publication history for this paper can be accessed here:

http://www.biomedcentral.com/1471-230X/12/178/prepub
